# Appraisal of different ultrasonography indices in patients with carotid artery atherosclerosis

**DOI:** 10.17179/excli2017-232

**Published:** 2017-05-17

**Authors:** Mehravar Rafati, Elham Havaee, Hassan Moladoust, Mohammadreza Sehhati

**Affiliations:** 1 Department of Medical Physics and Radiology, Faculty of Paramedicine, Kashan University of Medical Sciences, Kashan, Iran; 2 Cardiovascular Research Center, Guilan University of Medical Sciences, Rasht, Iran; 3 Department of Biomedical Engineering, School of Advanced Technologies in Medicine, Isfahan University of Medical Sciences, Isfahan, Iran

**Keywords:** carotid intima-media thickness, diagnostics, image processing software, mean wavelet entropy, statistical, ultrasonography

## Abstract

In this study a semi-automated image-processing based method was designed in which the parameters such as intima-media thickness (IMT), resistive index (RI), pulsatility index (PI), dicrotic notch index (DNI), and mean wavelet entropy (MWE) were evaluated in B-mode and Doppler ultrasound in patients presenting with carotid artery atherosclerosis. In a cross-sectional design, 144 men were divided into four groups of control, mild, moderate and severe stenosis subjects. In all individuals, far wall IMT, RI, PI, DNI, and MWE of the left common carotid artery (CCA) were extracted using the proposed method. Our findings showed that the maximum far wall IMT, RI, PI, DNI in the CCA were significantly different in the patients with mild, moderate, and severe stenosis compared to control group (p-value < 0.05), however, there were no significant differences in MWE among the four groups (p-value > 0.05). The proposed method can help physicians to better identify patients at risk of cardiovascular diseases.

## Introduction

Cardiovascular and cerebrovascular diseases are the leading causes of death and disability in the world. Our understanding of their etiology can be completed with a proper definition of the complicated pathophysiology and epidemiology of atherosclerosis (Stein et al., 2008[37]). Evaluations of atherosclerosis process by considering the arterial wall and blood flow velocity changes, may increase the chance for early diagnosis before revealing the clinical symptoms of cardiovascular diseases (CVD) and cerebrovascular diseases (Holewijn et al., 2010[14]). Common carotid intima-media thickness (IMT) assessed by B-mode ultrasound has been shown to have great reproducibility and can provide a reliable index for carotid atherosclerosis (Stein et al., 2008[37]). Besides, Doppler modality provided an appropriate tool to evaluate vascular flow velocity and systemic CVD in order to diagnosis and management of atherosclerotic conditions (Salk et al., 2014[35]). 

For evaluation of atherosclerosis situation within the arteries, five Doppler indicators in the common carotid artery (CCA) are used, viz., peak systolic velocity (PSV), end diastolic velocity (EDV), mean flow velocity, peak diastolic velocity (VD), and velocity of incisura between systole and diastole wave (VI). Three parameters were extracted from flow velocities: (i) resistive index (RI), (ii) pulsatility index (PI), and (iii) dicrotic notch index (DNI); they were suggested by Lee et al. for solving the problems caused by alterations in technique or apparatus (Lee et al., 2008[23]). These indices are important hemodynamic parameters that have been simply, accurately, and reproducibly used in the prognosis of cardiovascular complications (Staub et al., 2006[36]). Another common technique, which has been well applied to the study of ultrasound signals, is Fourier transform (FT) that is limited by the stationarity and linearity of system (Agnew et al., 2009[1]). The wavelet transform (WT) is a time-frequency representation that has an optimal resolution both in the time and frequency domains, and can overcome the limitations. Besides, entropy is a physical measure extracted from thermodynamic to explain the order/disorder of a dynamical system (Rosso et al., 2006[33]).

Most researchers have manually evaluated the arterial properties (Kumar and Nagarajan, 2015[19]; Williamson et al., 2008[41]), which is both time consuming and unreliable due to their dependency on individual assessment. Some studies have suggested an automated algorithm for measurement of arterial properties that is preferable (Kumar and Nagarajan, 2015[19]). Automated algorithm reduces the time of image processing and increases the reproducibility of the results (Kumar and Nagarajan, 2015[19]; Puchner et al., 2008[28]; Rafati et al., 2014[29]), however, the final results are not reliable in some cases. According to the above-mentioned problems, in this study semi-automated methods were designed and tested to evaluate the significance of diagnostic indices such as IMT, RI, PI, DNI, and mean wavelet entropy (MWE) in patients presenting with carotid artery atherosclerosis.

## Materials and Methods

### Signal analysis algorithms 

#### Modified Geometric Method (MGM)

An envelope detection algorithm, called the MGM, has been used over the Doppler signals within the carotid artery (Fernando et al., 2004[10]; Marzban et al., 2013[21]; Moraes et al., 1995[26]). In this method, the line connecting the maximum point and the minimum point of the curve is subtracted directly from the integral power spectrum curve, and the position of the maximum in the curve (envelope) is estimated as the maximum frequency (Fernando et al., 2004[10]; Marzban et al., 2013[24]). Normally, this technique is used on the signal's spectrum that was determined using Fast Fourier transformation (FFT). In this study, the same method was applied on the 2D image intensity values to determine the envelope of the blood flow velocity waveform curve. Three phases are part of this process, which brings about the identification of the envelope's point at each column of the image matrix. At the first stage, all intensity values in column direction were added up obtaining a cumulative distribution curve according to equation 1.


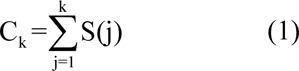


where *S(j)* is the intensity of the pixel in the current column and *j^th^* row and *C_k_* is the values of cumulative intensity curve for *k^th^* row. At the second stage, similar to MGM method, a linear function of row number with the same total intensity was defined, as given in equation 2.


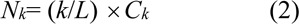


where *N_k_* is the uniform distribution of intensity of each column and *L* is the length of the column. Finally, the maximum of *C_k_ - N_k_* function was determined as the envelope candidate point as given in equation 3.


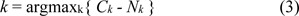


#### Mean Wavelet Entropy (MWE)

Discrete Wavelet Transform (DWT) is an efficient tool to assess different signals and images due to the inherent multiresolution features of the wavelet that preserve high and low frequency characteristics, which maintains the peaks and valleys that are usually present in curves. The MWE is a DWT-based measure of waveform complexity obtained from the Doppler signal's spectral content (Işler and Kuntalp, 2007[17]). This study involved obtaining measurement from the envelope of blood velocity waveform, which achieved by the MGM. Here, the envelope signal divided into six levels, using the mother wavelet of 'Daubechies 8', which is considered to be appropriate for Doppler signals (Işler and Kuntalp, 2007[17]). There were four phases at which the MWE was computed: first, relevant functions given by the MATLAB software were used to determine the wavelet packet coefficients. After getting to know the value of the wavelet coefficients, equation 4 was used to determine the energy for every coefficient, in which *C_j_* refers to the *j^th^* coefficient of the wavelet packet, while *K* refers to the number of levels. Then, the overall energy (*E_total_*) was calculated using equations 5 and 6 to signify the normalized wavelet energy (*N_E_*). Finally, equation 7 was used to explain the MWE, according to the Shannon entropy definition:


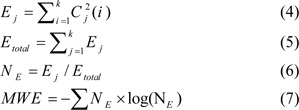


#### Resistive index (RI), Pulsatility index (PI), and Dicrotic notch index (DNI)

**Figure 1:**
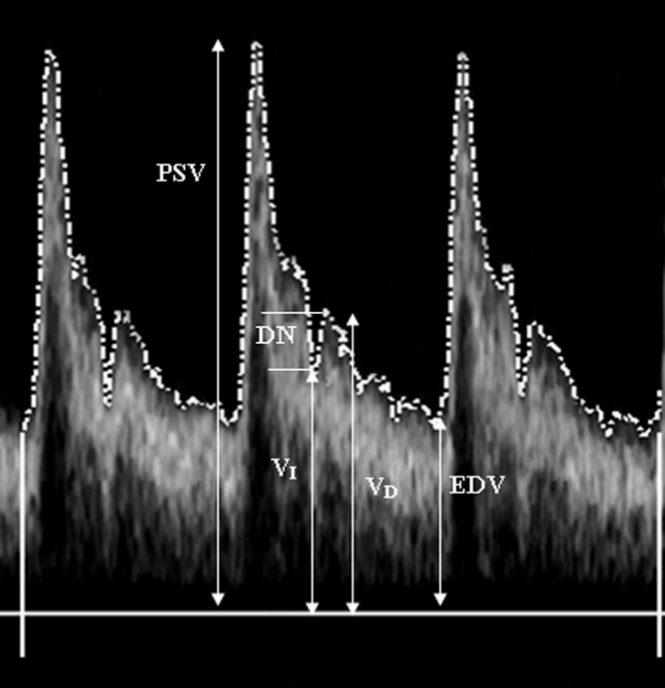
Envelope obtained from blood flow velocity recorded from a carotid artery with Modified Geometric Method (MGM). PSV, Peak Systolic Velocity; EDV, End Diastolic Velocity; dashed line, time averaged mean maximum velocity across the entire envelope of cardiac cycle (Vmean); V_D_, peak diastolic velocity; V_I_, velocity of incisura between systole and diastole wave; DN, dicrotic notch.

Doppler sonography can be used to obtain the RI and PI hemodynamic indices, which are evident from vascular resistance (Rafati et al., 2015[30]). Equation 8 was used to compute the RI.


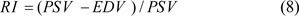


where, PSV signifies the peak systolic velocity, while EDV refers to the end diastolic velocity (Figure 1(Fig. 1)). Equation 9 was used to determine the PI.


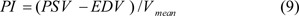


where, V_mean_ refers to the time averaged mean highest velocity over the complete envelope of the cardiac cycle.

The dicrotic notch (DN) refers to a declining wave which starts immediately after the peak systolic pressure and signifies closure of the aortic valve and the start of the diastolic stage. Alternations need to be measured as the velocity envelope waveform that is formed from atherosclerosis disease. The DNI signifies a hemodynamic index that is deduced from the difference between velocity of the bottom of the dicrotic notch (DN) and the first peak in the diastolic velocity by employing the Doppler sonography. Equation 1) was used to obtain the DNI.


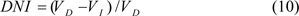


where, V_D_ refers to the peak diastolic velocity and V_I_ refers to the velocity of incisura between diastole and systole wave.

### IMT measurement 

This study involves the application of a latest technique to measure IMT changes in the CCA during three cardiac cycles (Gustavsson et al., 1997[13]; Jegelevicius and Lukosevicius, 2002[18]; Molinari et al., 2012[25]). The method used dynamic programming and maximum gradient algorithm to determine the reference points and the cost function, respectively. This semi-automatic method was comprehensively introduced and evaluated on ultrasound imaging of the carotid artery giving best results in our previous studies (Rafati et al., 2014[29], 2015[30]).

### Study population

A cross-section design involved performing an analysis in which 144 men in the age group of 40 to 60 years participated. This study was carried out from April 2016 to October 2016. Four groups were formed, consisting of a control group (that did not have any history of cardiovascular and/or cerebrovascular disease, hypercholesterolemia, diabetes mellitus, hypertension and tobacco abuse) (Tendera et al., 2011[38]), mild stenosis (< 50 %), moderate stenosis (50 %-69 %), and severe stenosis (> 70 % stenosis) (Grant et al., 2003[12]; Leclerc et al., 1999[22]). Angiography and Doppler were used to assess the carotid stenosis. Angiography allowed for determining the extent of stenosis by evaluating the diameter of the residual lumen at its narrows point and the diameter of the distal internal carotid artery (ICA) at the point at which it was believed to be free of disease, preventing calcification and a typical thickness of the wall. No measurement was done when ICA appeared to be normal (Grant et al., 2003[12]; Leclerc et al., 1999[22]). In terms of Doppler, there were characterizations, like PSV of ICA < 125 cm/s, ICA/CCA PSV < 2, EDV of ICA < 40 cm/s, whereas there was no evident plaque or intimal thickness in the sonography performed on the control group. The mild group and the control group depicted the same Doppler characterizations; however, the plaque or intimal thickening was evident via sonography in the mild group. In the moderate group, Doppler characteristics were determined as: ICA PSV=125-230 cm/s, ICA/ CCA PSV=2-4, ICA EDV=40-100 cm/s, whereas plaque was evident. Lastly, the following characteristics of Doppler were evident in the severe group: ICA PSV > 230 cm/s, ICA/CCA PSV > 4, end ICA EDV > 100 cm/s, while plaque or luminal narrowing was evident at gray scale and Doppler ultrasound (Grant et al., 2003[12]; Leclerc et al., 1999[22]). It was confirmed in the angiography that there was considerable stenosis in the left bulb and ICA.

### Ultrasound studies and offline analysis

Prior to performing ultrasonography on the selected sample, every participant was requested to rest in the supine position until their heart rate and blood pressure get back normal. The blood pressure and heart rate were measured using an oscilloscopic blood manometer and also by using a wrist manometer at the sites where brachial and radial arteries are located. The recordings of both blood pressure and the heart rate were taken in a controlled room temperature as given in the instruction manual (Onbaş et al., 2007[27]). CCA of the left side of the selected population was checked 2-3 cm before the bifurcation of the artery using Sonoline Antares ultrasound systems having 5-13 MHz of linear transducer (Siemens, Germany). All measurements were performed by an expert-trained sonographer in radiology department at Beheshti hospital that is associated with Kashan University Medical Sciences, located in Kashan, a city of Iran. The dynamic range, gray level and the depth of focus were assessed to be 55 dB, 0 to 255, and 3.5 cm, respectively. In the final stage, the rate at which the blood flows from the mid-lumen of the arteries was taken out using color Doppler sonogram of the left CCA, categorizing in four groups. Doppler flow signals were set for an insonation angle at 60 degrees. The signals were recorded at the rate of 30 frames per second by personal computer using audio-video interleaves (AVI) format. Every recording composed of a total of three cardiac cycles, which is 90 frames, taken in longitudinal direction of the left CCA. For offline analysis, we used the proposed algorithm for recording the instantaneous alternations on the carotid IMT during three cardiac cycles that were averaged from maximum IMT in the far wall CCA (Rafati et al., 2014[29], 2015[30]). Besides, two other programs were designed in Matlab software; one for extraction of PSV, EDV, V_mean,_ V_D_, and V_I_ and the other program for calculation of MWE from the blood flow velocity curve. The measurements from three cardiac cycles were averaged before further analysis. Measurements of electrocardiogram (ECG), blood velocity, diameter, and IMT were synchronized while evaluating the CCA. 

### Statistical analysis

For analyzing the data statistically, the software SPSS version 13.0 (SPSS Inc. Chicago, IL, USA) was used. A paired t-test analysis was used to assess the differences in the values obtained by two methods. Pearson's correlation coefficient and linear regression between the outcomes of the proposed and the manual methods were evaluated. The associations between the two methods were re-evaluated according to the analysis of Bland-Altman. Intra-observer variability suggested by an expert sonographer and inter-observer variability suggested by two expert sonographers showed that 20 of the participants showed as a percentage of the error of the means. All examinations were performed under the same standard conditions; and the percentage of coefficient of variation (%COV) was reported to show the efficiency and reproducibility in each experiment. The data were examined for normal distribution and homogeneity of variance by the K-S and Levene test, respectively. ANOVA tests were applied to examine significant differences in values of IMT, RI, PI, V_D_, V_I_, and MWE. Then, Bonferroni was used to process the post hoc test. A p-value of < 0.05 was considered as statistically significant in all steps of the analysis. 

## Results

At the first stage, the ultrasonic examination of the left CCA of twenty human subjects (aged 40-60 years) was performed, who do not have an earlier problem related to cardiovascular and/or cerebrovascular disease, hypertension, and diabetes mellitus and tobacco abuse. The PSV, EDV, V_mean_, V_D_, and V_I_ were calculated by the given algorithm from the blood flow velocity recorded from the CCA during three cardiac cycles in all 20 subjects (Table 1(Tab. 1)). 

**Figure 2:**
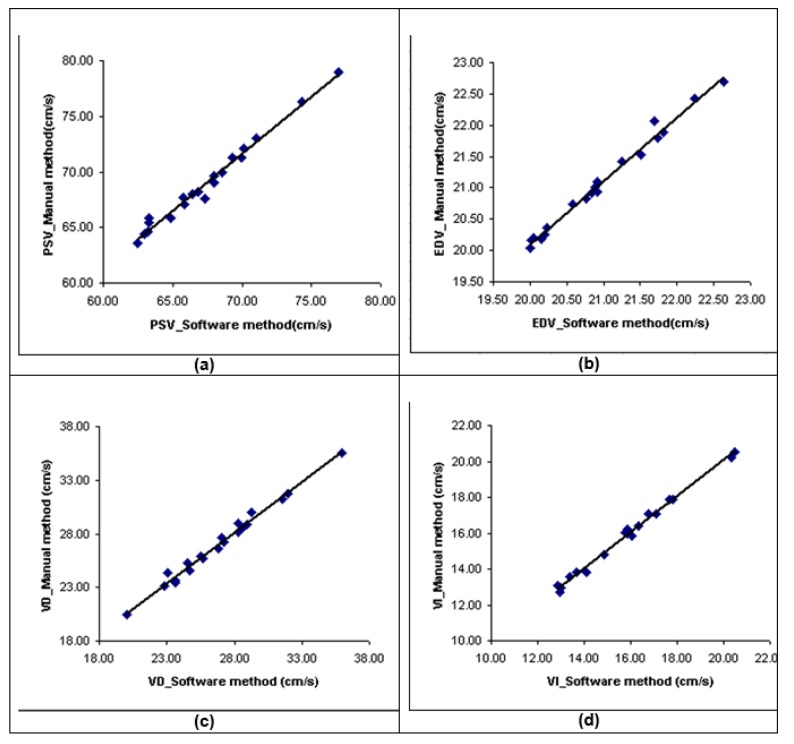
Figure 2a, b, c and d) Scatter plot demonstrating the correlations for PSV, EDV, V_D_, and V_I_ measurements between proposed computerized analysis and manual tracing

The results of the statistical analysis demonstrated that no significant difference was found between the given and manual procedures (p > 0.05). There was a significantly high correlation between the velocities measured by both proposed and manual tracing methods (r=0.98, p < 0.001 for PSV, EDV, V_D_, and V_I_) (Figure 2a-d(Fig. 2)).

By making use of linear regression analysis, the regression function between PSV, EDV, V_D_, and V_I_ measured by manual tracing and the proposed method was predicted, respectively as equations 11-14.


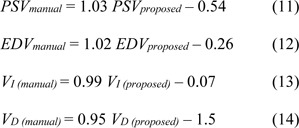


**Figure 3:**
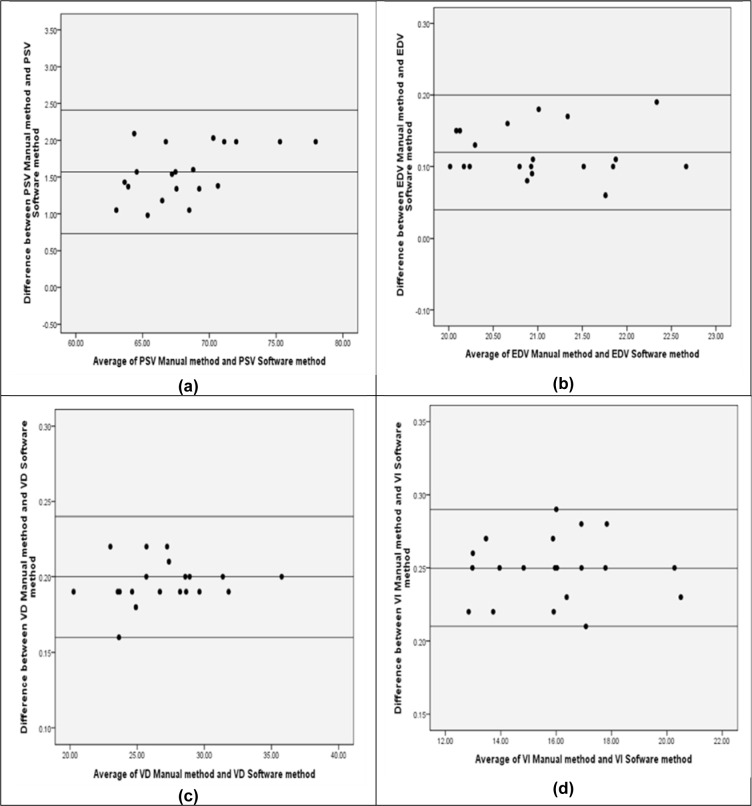
Figure 3a, b, c and d) Relative Bland-Altman plot of differences between the methods for PSV, EDV, V_D_, and V_I_. The outer lines represent 1.96 SD or 95 % limits of agreement (LOA).

For Bland-Altman analysis, the difference between estimated velocity changes and the middle line indicates the average difference between the two methods, where the outer lines represent 1.96 SD or the 95 % limits of agreement (LOA) (Figure 3 a-d(Fig. 3)). 

There was appropriate LOA and considerably high agreement between the two methods. The mean difference for measured PSV, EDV, V_D_, and V_I_ were 1.57 ± 0.42 cm/s, 0.12 ± 0.04 cm/s, 0.20 ± 0.04 cm/s, and 0.25 ± 0.04 cm/s, respectively. Intra-observer and inter-observer variabilities in PSV were found to be 0.19 % and 0.23 % for semi-automated methods while they were 0.26 % and 0.29 % for manual methods. These four variabilities were found to be 0.30 %, 0.34 %, 0.40 % and 0.46 % for EDV; 0.52 %, 0.72 %, 0.65 and 0.79 % for V_D_; and 0.43 %, 0.51 %, 0.59 and 0.65 % for V_I_, respectively. In the proposed method, the coefficient of variation (percent COV) in PSV, EDV, V_D_, V_I_ were 5.65 %, 3.65 %, 6.25 %, and 6.93 %, respectively. The COVs in PSV, EDV, V_D_, and V_I_ for manual tracing were 5.77 %, 3.74 %, 6.47 %, and 7.65 %, respectively.

In the second step, the summaries of major demographic factors of the four groups of subjects are given in Table 2(Tab. 2). The statistical analysis of age and body mass index (BMI) showed that there are no significant differences among the four groups; p-values are 0.08 and 0.20, respectively. The heart rate (HR), systolic and diastolic blood pressure (P_s_ and P_d_, respectively) in mild, moderate and severe stenosis groups are greater than those in control group. However, the variations are not significant in the four groups; p-values are 0.25, 0.27 and 0.70, respectively. 

**Figure 4:**
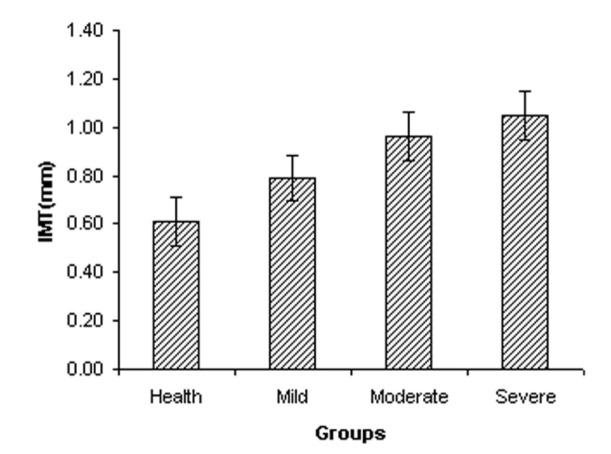
The maximum far wall IMT (mm) in left common carotid artery in each group

The measured values of maximum far wall IMT are presented in Figure 4(Fig. 4). The IMT of the left CCA was progressively increased from 0.61 ± 0.10 mm in the control subjects to 0.79 ± 0.09 mm in the mild stenosis group, to 0.96 ± 0.10 mm in the moderate stenosis group and 1.05 ± 0.10 mm in the severe stenosis group, respectively. Figure 4(Fig. 4) showed that maximum far wall IMT in the left CCA is significantly increased with the progress of atherosclerosis (p-value < 0.05). The COVs of maximum far wall IMT were 16.73 %, 11.57 %, 10.28 % and 9.78 % for control, mild stenosis, moderate stenosis and severe stenosis, respectively. The RI of the left CCA increased from 0.66 ± 0.05 in the control subjects to 0.77 ± 0.05 in the mild stenosis group, to 0.86 ± 0.05 in the moderate stenosis group and 0.89 ± 0.06 in the severe group. RI of CCA was significantly different between the control and three other groups (p-value < 0.05). The COVs of RI were 7.53 %, 6.48 %, 5.81 % and 6.38 % for control, mild stenosis, moderate stenosis, and severe stenosis, respectively.

**Figure 5:**
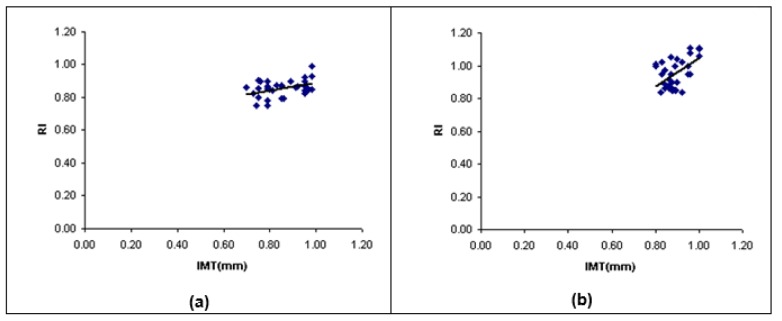
Scatter plot between IMT and RI of the LCCA in (a) moderate stenosis group and (b) severe stenosis group. LCCA, left common carotid artery; IMT, intima-media thickness; RI, resistive index; R, correlation efficient

The statistical correlation between maximum far wall IMT and RI of the left CCA was estimated. There was no significant correlation between two parameters in control and mild groups (r=0.13, r=0.24, respectively; p-value > 0.05). Figure 5a, 5b(Fig. 5) showed that there was a small significant correlation between two parameters in moderate stenosis group (r=0.42, p-value < 0.05), and there was significant correlation between two parameters in severe stenosis group (r=0.53, p-value < 0.05). The linear regression function was predicted via a linear regression analysis (Figure 5a, 5b(Fig. 5)).

The PI of the left CCA increased from 1.34 ± 0.05 in the control subjects to 1.44 ± 0.10 in the mild stenosis group, to 1.56 ± 0.07 in the moderate stenosis group and 1.80 ± 0.12 in the severe group. PI of CCA was significantly different in the patients with mild, moderate and severe stenosis compared to control group (p-value < 0.05). The COVs of PI were 9.12 %, 4.00 %, 4.69 % and 6.75 % for control, mild stenosis, moderate stenosis, and severe stenosis, respectively.

**Figure 6:**
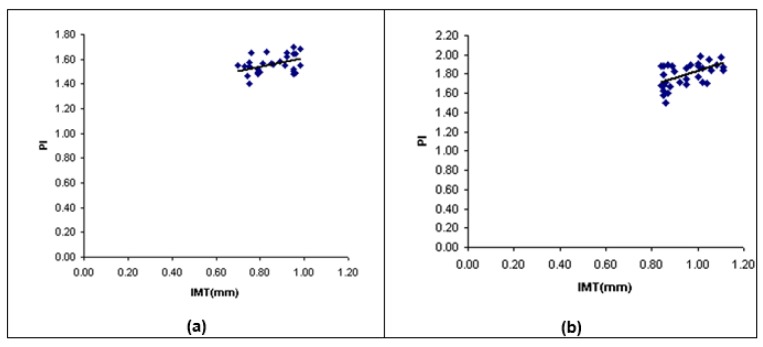
Scatter plot between IMT and PI of the LCCA in (a) moderate stenosis group and (b) severe stenosis group. LCCA, left common carotid artery; IMT, intima-media thickness; PI, pulsatility index; R, correlation efficient

The statistical correlation between maximum far wall IMT and PI of the left CCA was estimated. There was no significant correlation between two parameters in control and mild groups (r=0.18, r=0.28, respectively; p-value > 0.05). Figure 6a, 6b(Fig. 6) showed that there was mild significant correlation between two parameters in moderate stenosis group (r=0.45, p-value < 0.05), and there was significant correlation between two parameters in severe stenosis group (r=0.55, p-value < 0.05). The linear regression function was predicted via a linear regression analysis (Figure 6a, 6b(Fig. 6)). 

**Figure 7:**
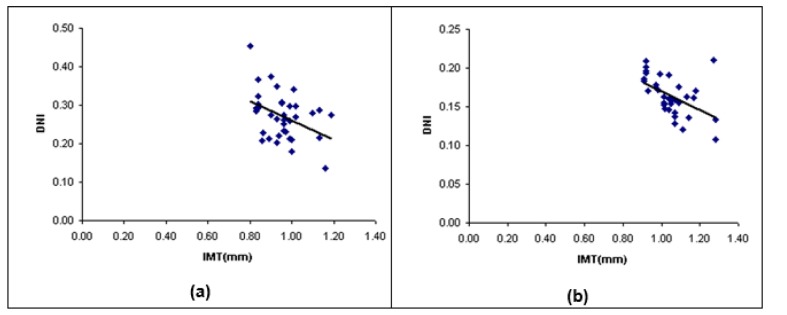
Scatter plot between IMT and DNI of the LCCA in (a) moderate stenosis group and (b) severe stenosis group. LCCA, left common carotid artery; IMT, intima-media thickness; DNI, dicrotic notch index; R, correlation efficient

The DNI of the left CCA was sequentially decreased from 0.41 ± 0.05 in the control subjects to 0.31 ± 0.03 in the mild stenosis group, to 0.27 ± 0.03 in the moderate stenosis group and 0.16 ± 0.02 in the severe group. DNI of CCA was significantly different in patients with stenosis compared to control group (p-value < 0.05). The COVs of DNI were 12.13 %, 9.74 %, 9.94 % and 9.17 % in four groups, respectively. The statistical correlation between maximum far wall IMT and DNI of the left CCA was estimated. There was no significant correlation between two parameters in the control and mild groups (r=-0.15, r=-0.27, respectively; p-value > 0.05), Figure 7a, 7b(Fig. 7) showed that there was a small significant correlation between two parameters in the moderate stenosis group (r=-0.40, p-value < 0.05), and there was significant correlation between two parameters in the severe stenosis group (r=-0.50, p-value < 0.05). The linear regression function was predicted via a linear regression analysis (Figure 7a, 7b(Fig. 7)).

The MWE of the left CCA was sequentially decreased from 1.15 ± 0.11 in the control subjects to 1.12 ± 0.14 in the mild stenosis group, to 1.09 ± 0.11 in the moderate stenosis group and 1.07 ± 0.16 in the severe group. There was no significant differences in MWE of CCA between the control group and other groups (p-value > 0.05). The COVs of MWE were 9.35 %, 12.83 %, 10.37 %, and 15.02 % in four groups, respectively.

## Discussion

Many non-invasive decision makers are related to the early variations occur in vessel walls which includes expansion of arterial wall, endothelial dysfunction, and coronary artery calcification (Coll and Feinstein, 2008[8]; Holewijn et al., 2010[14]). There are a number of such processes, including the carotid IMT measurement that is a non-ionizing risk, non-invasive and reproducible technique for discriminating and quantifying sclerotic arteries (Hurst et al., 2007[15]). Evaluation of the far wall of CCA by using the B-mode ultrasound has important advantages over ICA and bulb. Its size, relatively superficial position, ease accessibility, and limited movement provided a proper situation for studying carotid structure in this portion of carotid artery (Stein et al., 2008[37]). Moreover, the far wall gives much better reflections because of the interface of blood-intima and intima-media layers for more convenience (Laurent et al., 2006[21]). 

According to the previous results, which were obtained by Rodriguez-Hernandez et al. (2003[32]) in untreated hypertensive patients, there is an important difference between IMT of the left and right side CCA. They noticed that IMT value is higher in the left than in the right CCA. Possible tendency for cerebrovascular diseases in the left may be because of increased intimal hyperplasia or more extensive medial hypertrophy; the two conditions can develop later on as increased hemodynamic stress at the side (Onbaş et al., 2007[27]; Rodríguez Hernández et al., 2003[32]). There are other researches that ensure the results of a significantly higher CCA-IMT on the left side, but it gives different results in blood pressure, shear stress, and vascular anatomy between the two sides (Foerch et al., 2003[11]). 

According to a more recent study, (Rafati et al., 2015[30]), it showed that high resolution B-mode ultrasound can determine the properties of artery during the cardiac cycle using a computerized analysis system. The various algorithms were calculated and then comparison was made for IMT measurement. The earlier evaluations all used a computerized analytical process to identify lumen diameter and IMT in a single frame, and methods to give the actual dimensions over the entire cardiac cycle to capture the mechanical nature of the artery. The current research evaluated maximum far wall IMT of the left CCA of 144 men using a semi-automated method in the four groups of subjects.

There was a significantly increase in maximum far wall IMT in the control group compared to the severe stenosis group (p-value < 0.05), which is in agreement with the earlier researches (Baldassarre et al., 2012[4]; Crouse et al., 1994[9]). It may be due to intimal layer migration/proliferation of vascular smooth muscle cell, sub-endothelial collagen, and collagen cross-linking, elastin fragmentation, and proteoglycan function increased wherever endothelial permeability improved, endothelial nitric oxide values reduced, endothelin function increased, inflammatory markers values improved, and superoxide dismutase activity decreased with vascular stiffness and atherosclerosis process (Chow et al., 2013[7]; Wagenseil and Mecham, 2012[40]). Blood flow velocity via time graphic based on ultrasound technique is a popular, safe, and non-invasive tool to assess arterial effect of atherosclerosis in CVD and cerebrovascular diseases. It gives essential information about the presence of disturbed flow in sclerotic carotid artery (Rubins et al., 2012[34]). The RI and PI are the most practical parameters for quantifying changes in the CCA blood velocity waveforms. Both of these are ratios that are dependent on the waveform shape and independent from the angle of insonation (Azhim et al., 2007[3]). 

The variabilities associated with inter-observer and intra-observer of IMT was higher than those of RI and PI (Staub et al., 2006[36]). However, when compared to the earlier researches, in this research, we found a greater correlation in between RI, PI and IMT in the CCA (Baldassarre et al., 2012[4]; Crouse et al., 1994[9]; Lee et al., 2008[23]). This was probably due to the elevated circumferential wall tension that made fast travelling pulse waves which can cause the arteries to become stiffer as compared to the greater systolic blood pulse (Chow et al., 2013[7]). Another reason why the arteries can become stiff is the increase in the RI and PI which may be due to reduction in the speed of blood flow. These given factors are the cause of elevated harm to arterial wall along systematic atherosclerosis and can also reduce shear stress (Chow et al., 2013[7]). In addition to RI and PI, magnitude of arterial elastic recoil during diastolic phase affected from damaged muscular tone can be also evaluated by DNI. We found a significant reduction in DNI in the control group as compared to the severe stenosis group. We also found a significant correlation in the maximum IMT and DNI in both groups (Moderate and Severe stenosis). This is because of hemostical hardness related to the elevated stiffness of arteries and decreased elasticity with growth of atherosclerosis (Ilea et al., 2013[16]). In addition, the COVs of RI, PI, and DNI were less than IMT in all groups which may be due to better reproducibility of them.

Because of the methodologies introduced and applied in this work, inter-observer and intra-observer variabilities in our results were found to be less than the manual tracing method. In addition, the proposed MGM algorithm has some advantages compared with the manual method or the common algorithms of blood velocity envelope extraction, such as envelope detection based on the threshold method that was used in some ultrasound devices. First, the MGM is a robust algorithm that can be adapted properly for different signal to noise ratios (SNR), and presents the same values of standard deviation (SD) when compared with other processes (Fernando et al., 2004[10]; Moraes et al., 1995[26]). Following this, the usage of 2D image intensity values for obtaining envelope of the blood velocity wave led to simplicity of computation and efficiency in the ultrasound image recognition. These aspects make the proposed approach more suitable for usual software aims in various Doppler systems.

The researchers working on MWE measure in the modern medical applications have worked on its capabilities for identification of cardiac arrhythmia, atrial fibrillation, changes of electrocardiogram (ECG) or electroencephalogram (EEG) signals, and see whether it is even suitable for any pre-clinical evaluation of disturbed blood flow velocity shapes such as othalmic and common carotid arteries issues in patients (Alcaraz and Rieta, 2012[2]; Bleakley et al., 2015[5]; Işler and Kuntalp, 2007[17]; Langley, 2015[20]; Ródenas et al., 2015[31]; Rosso et al., 2006[33]). The researchers have emphasized that MWE has some benefits like short computational time, robustness against noise, parameter-free and detecting changes in non-stationary signal because it applies wavelet transform in multi-resolution foundation. In the current study Daubechies-8 mother wavelet was used for measuring MWE values. The selection of a suitable mother wavelet is very crucial for extracting information from data, and useful mathematical properties of Daubechies including orthogonal nature for preserving energy and more ability for overlapping windows helped us to extract all changes among pixel intensities. This property makes better localization and smoothness compared with other mother wavelets (Chaudhary and Dhamija, 2013[6]). Regarding the above-mentioned aspects, the MWE values were measured in four groups using our proposed semi-automated method on the CCA during three cardiac cycles. The results showed a non-significant (p-value > 0.05) decrease from control group to severe stenosis group in terms of MWE values in the CCA that is probably because of lack of nitric oxide (NO) released from endothelium of arterial wall during atherosclerosis development (Vicenzini et al., 2007[39]). This is due to the correlation of MWE parameter with the variations found in the energy of blood pressure and flow velocity signals in smaller arteries rather than large arteries (Bleakley et al., 2015[5]).

## Conclusion

Semi-automated method for extraction and significance analysis of hemodynamic parameters and MWE in 144 Iranian males with carotid artery atherosclerosis has been evolved. We conclude that the IMT, RI, PI, and DNI of the left CCA are proper parameters for morphological and functional evaluation of carotid atherosclerosis development. This information about hemodynamic artery helps physicians to better classify patients at risk of CVD and stroke incidence.

Although attempts were made to decrease movements, complications created during scanning and reduction of artery pulsating movement by the translation movement of the CCA originated from the probe appear to be a limitation of this study. The data were gained for this study from the Iranian male population which is another restriction of this study; hence it is controversial whether these results can be hypothesized for the gender/ethnic groups.

## Acknowledgements

We would like to thank Dr. Kamran Hami and Yaser Hamedian from radiology department Beheshti Hospital at Kashan University of Medical Sciences. This research did not receive any specific grant from funding agencies in the public, commercial, or not-for-profit sectors.

## Conflict of interest

The authors declare that they have no conflict of interest.

## Ethical approval

This study was approved by the ethics committees of Kashan University of Medical Sciences (Iran). All the subjects were given informed written consent prior to participation in the study.

## Figures and Tables

**Table 1:**
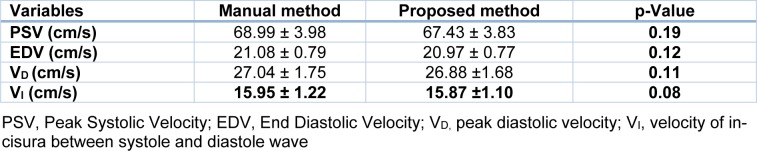
The mean ± SD of PSV, EDV, V_D_, and V_I_. The left CCA was extracted throughout three cardiac cycles with the proposed and the manual method.

**Table 2:**
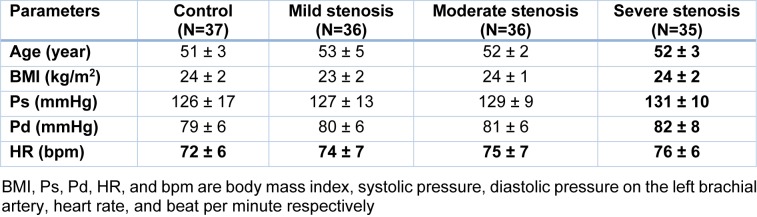
Main demographic characteristics (mean±SD) of four groups (control, mild stenosis, moderate stenosis and severe stenosis) subjects.
